# Effects of Xuezhikang versus Pravastatin on Triglyceride Level in Patients with T2DM and Dyslipidemia: Study Protocol for a Multicenter Randomized Controlled Trial

**DOI:** 10.2174/1570161121666230328110215

**Published:** 2023-08-01

**Authors:** Jin Xu, Liyuan Zhu, Yingying Xie, Miao Zhang, Zixi Xiao, Rongkai Su, Tie Wen, Ling Liu

**Affiliations:** 1 Department of Cardiovascular Medicine, The Second Xiangya Hospital, Central South University, Hunan, 410011, China;; 2 Research Institute of Blood Lipid and Atherosclerosis, Central South University, Hunan, 410011, China;; 3 Modern Cardiovascular Disease Clinical Technology Research Center of Hunan Province, Hunan, 410011, China;; 4 Cardiovascular Disease Research Center of Hunan Province, Hunan, 410011, China;; 5 Department of Emergency Medicine, Second Xiangya Hospital, Central South University, Hunan, 410011, China;; 6 Emergency Medicine and Difficult Diseases Institute, Second Xiangya Hospital, Central South University, Hunan, 410011, China

**Keywords:** Diabetes, triglyceride, dyslipidemia, hypertriglyceridemia, cardiovascular risk, diabetes

## Abstract

***Background*:** Hypertriglyceridemia, is commonly found in patients with diabetes. Xuezhikang, an extract of red yeast rice, is effective in reducing cardiovascular events in Chinese patients with diabetes and coronary heart disease (CHD). Xuezhikang has been reported to significantly decrease the level of triglycerides (TG), a potential causal risk factor for myocardial infarction. On the basis of a similar reduction in low-density lipoprotein cholesterol, this study will evaluate the effect of xuezhikang on TG levels compared with pravastatin in patients with type 2 diabetes mellitus (T2DM) and dyslipidemia.

***Methods*:** This is an open-label, multicenter, randomized controlled study to assess the effects of xuezhikang (1.2 g/day) and pravastatin (20 mg/day) on TG and other blood lipid parameters in patients with T2DM and dyslipidemia. A total of 114 patients will be enrolled and randomly assigned 1:1 to receive xuezhikang or pravastatin treatment for 6 weeks.

***Results*:** The primary outcome measure is the change from baseline in fasting TG levels after 6 weeks. The change from baseline in other fasting and postprandial lipid parameters, and glucose profiles at 1, 2, and 4 h after a nutritious breakfast will also be explored.

***Conclusion*:** This study will evaluate the effect of a 6-week treatment with xuezhikang compared with pravastatin on fasting and postprandial TG levels and other blood lipid parameters in patients with T2DM and dyslipidemia without atherosclerotic cardiovascular disease (ASCVD). The results will provide more information on optimizing the lipid control of patients with diabetes in the primary prevention of ASCVD.

***Trial Registration*:** Chinese Clinical Trial Registry, ChiCTR2100046704. Registered 27 May 2021.

## 1 INTRODUCTION

Type 2 diabetes mellitus (T2DM) is an independent risk factor of atherosclerotic cardiovascular disease (ASCVD) [1, 2], and has been regarded as an equivalent risk of coronary heart disease (CHD). Diabetes is often associated with a particular lipid spectrum, characterized by an increase in triglycerides (TG) and small dense low-density lipoprotein cholesterol (LDL-C), and a decrease in high-density lipoprotein cholesterol (HDL-C) [3], which makes treating diabetes more complicated. For patients with T2DM, controlling LDL-C levels is the first goal of lipid-lowering therapy for both primary and secondary prevention [4]. Elevated TG levels, especially in the postprandial state, increases ASCVD risk for patients with diabetes [5, 6]. The second goal is to control non-HDL-C levels, which is important for patients with diabetes [7]. Non-HDL-C includes the cholesterol content of nearly all atherosclerotic lipoproteins, including LDL, lipoprotein (a) [Lp(a)], and TG-rich lipoproteins and their remnants [8].

At present, statins are the cornerstone of cholesterol control in patients with diabetes [9]; however, the effects of statins on TG levels varied in previous studies. Rosuvastatin has been reported to reduce TG levels by 12-37% across a dose range of 5-20 mg/day [10]. The TG reduction of atorvastatin ranged from 11.6% for 2.5 mg/day to 32.15% for 80 mg/day [11]. Xuezhikang, a polypill extracted from red yeast rice, contains 13 kinds of natural statins, including lovastatin and its homologues, as well as unsaturated fatty acids, ergo sterol, flavonoids, and other active ingredients [12, 13]. Another study reported that non-statin elements of xuezhikang may have synergistic effects in improving blood lipids and inhibiting the synthesis of TG [14]. Xuezhikang was also effective in the reduction of cardiovascular events, including myocardial infarction and CHD death, in Chinese diabetic patients with CHD [15, 16]. Interestingly, xuezhikang significantly decreased fasting TG levels in addition to LDL-C in Chinese patients with T2DM [17]. Moreover, xuezhikang was shown to be beneficial in the treatment of postprandial hypertriglyceridemia after a high-fat meal in patients with CHD without T2DM [18]. Considering that high TG levels, especially in the postprandial state, are a causal risk factor for myocardial infarction [19], lowering TG levels may contribute to the reduction of ASCVD risk in patients with diabetes.

It has been reported that xuezhikang 1.2 g/day or pravastatin 20 mg/day reduced the LDL-C level by a similar extent, about 24-28% [12, 20-23]. Given that both xuezhikang and pravastatin reduce LDL-C, similarly, our study compares the clinical benefit and safety of both xuezhikang and pravastatin in patients with T2DM and dyslipidemia without ASCVD, in terms of TG reduction in the fasting and postprandial states.

## 2 MATERIALS AND METHODS

### 2.1 Study Design

This is an open-label, multicenter, prospective, randomized controlled study to evaluate the effects of xuezhikang (1.2 g/day) and pravastatin (20 mg/day) on TG levels and other blood lipid parameters for 6 weeks in patients with T2DM and dyslipidemia (study protocol version 4.0, dated 13 Jan 2021). Pravastatin is used as a comparator in this study as previous studies have shown that the magnitude of LDL-C reduction of pravastatin and xuezhikang at current dosages were similar [12, 20]. The clinical trial registration number (ChiCTR2100046704) can be found at http://www.chictr.org.cn.

Patients with T2DM and dyslipidemia will be enrolled and randomly assigned (1:1) to xuezhikang or pravastatin therapy in 3 centers in China. Sealed envelopes with a red card or a white card, representing the experimental group (xuezhikang) and the control group (pravastatin), respectively, will be used to allocate the treatment arm. Blood lipids at fasting and 1, 2, and 4 h after a nutritious breakfast will be monitored at baseline and 6 weeks after treatment. Only drugs affecting lipid metabolism will be prohibited during the trial. Treatment will be discontinued because of withdrawal by patient for any reasons or decision by the investigator for safety reasons.

### 2.2 Patient Population

Eligible patients must meet the following criteria before entering this study.

### 2.3 Inclusion Criteria

1. Aged ≥ 18 years.
2. Diagnosed with T2DM and dyslipidemia. T2DM should be diagnosed according to American Diabetes Association Standards of Medical Care in Diabetes (2020) [24]. Dyslipidemia should be diagnosed according to the test within 4 weeks, and both of the following conditions should be met: (1) Fasting TG ≥1.7 mmol/L and <5.6 mmol/L; (2) Fasting LDL-C ≥1.8 mmol/L and <4.9 mmol/L.
3. Patients should have at least one of the following risk factors: (1) T2DM duration ≥10 years; (2) Smoking; (3) Obesity (body mass index ≥28 kg/m^2^, or waist circumference ≥90 cm [male] or ≥85cm [female]); (4) Hypertension; (5) Fasting HDL-C <1.0 mmol/L or LDL-C ≥2.6 mmol/L within 4 weeks.

### 2.4 Exclusion Criteria

1. Known ASCVD including CHD, ischemic stroke, transient ischemic attacks, peripheral artery disease, etc.
2. Use of lipid-lowering drugs within 3 months prior to enrollment.
3. Uncontrolled diabetes with HbA1c ≥8.0%.
4. Active liver disease or dysfunction including sustained serum transaminase elevation of unknown reason or higher than three-fold of the upper limit of normal.
5. Known myopathy or serum creatine kinase >5-fold of the upper limit of normal not caused by muscle injury.
6. Contraindication to xuezhikang capsules, pravastatin, long-term use of glucocorticoids or contraceptives.
7. At the acute stage of infectious diseases, or with any one of the following diseases: hyperthyroidism, hypothyroidism, acute cerebrovascular disease, severe cardio-renal insufficiency (glomerular filtration rate <60 mL/min/1.73m^2^), malignant tumor, hematopoietic disease, autoimmune disease, digestive system disorders affecting digestion and/or absorptive function, mental disorder, other severe or unstable physical disease.
8. A history of alcohol or drug abuse or dependence within 3 months before inclusion in the trial.
9. Participation in clinical trials of other drugs within 3 months before inclusion in the trial.
10. Other conditions that make it inappropriate for participation at the investigator's discretion.

### 2.5 Enrollment Schedule

**Fig. (1):**
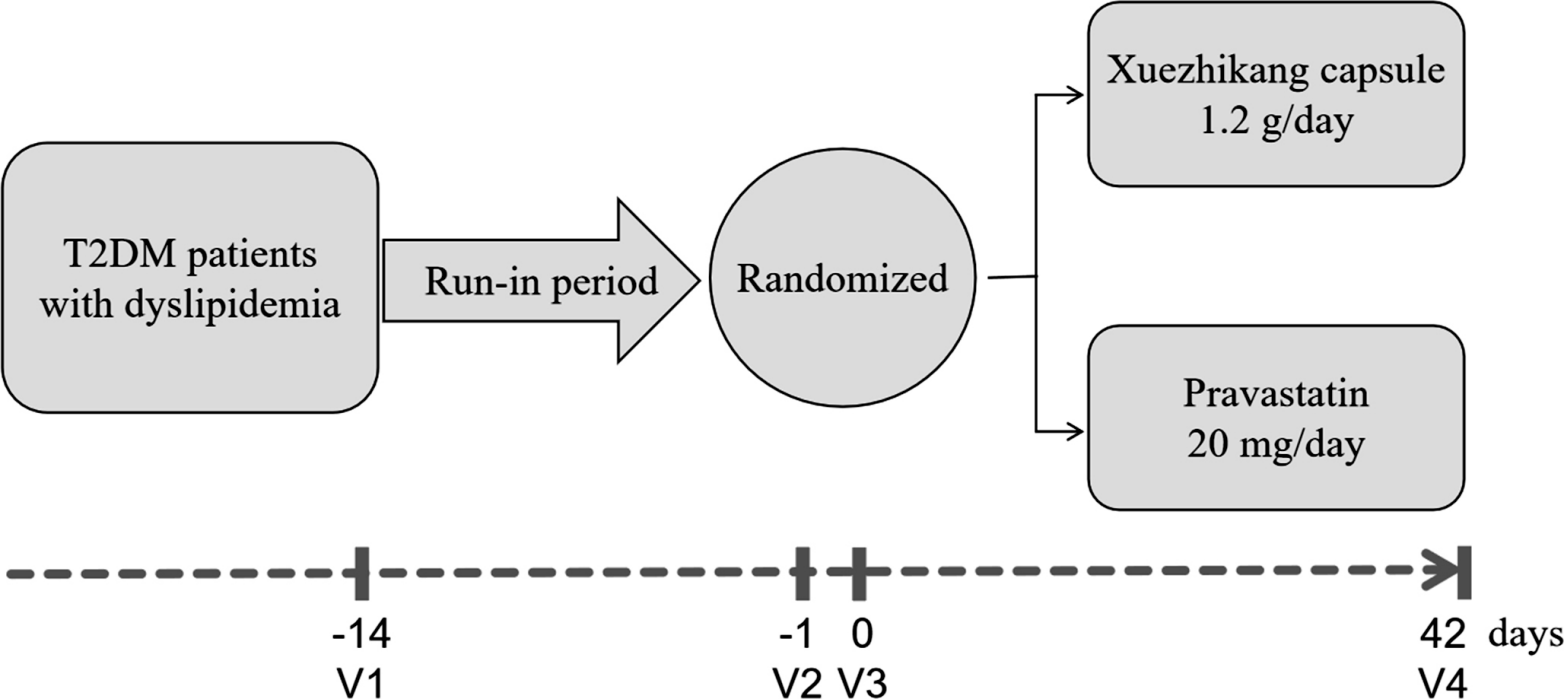
Study design. **Abbreviations:** T2DM, type 2 diabetes mellitus; V, Visit.

This study consists of four visits: screening period, run-in period, before and after the 6-week treatment (Fig. **1**). Patients will be screened for study eligibility (Visit 1). After screening, health education and diet instructions will be given to eligible patients. Briefly, they are suggested to follow a low-fat, low-salt diet, and avoid alcohol and strenuous exercise. Patients will be requested to follow the instructions for at least 14 days. No lipid-lowering drug is allowed during this period. After a 14-day run-in period, patients will receive blood lipid tests and safety assessments (Visit 2). Eligible patients will be randomized to receive xuezhikang or pravastatin. A nutritious breakfast with a total of 800 kcal will be given as described previously [12], followed by blood lipid tests at fasting and 1, 2, and 4 h after breakfast (Visit 3). Patients will be assessed again after 6 weeks of treatment. (Visit 4).

### 2.6 Outcome Measures

The primary outcome measure is the change from baseline in fasting TG levels after 6 weeks of xuezhikang (1.2 g/day) and pravastatin (20 mg/day). The secondary outcome measures are change from baseline after 6 weeks of treatment in other fasting lipid indexes (including total cholesterol, LDL-C, HDL-C, non-HDL-C, remnant cholesterol, Lp(a), apolipoprotein [Apo] A1, and ApoB), change from baseline in postprandial TG levels 1, 2, or 4 h after breakfast, fasting, and postprandial plasma glucose and insulin levels, fasting HbA1c level, and change from baseline after 6 weeks in fasting and postprandial levels of high-sensitivity C-reactive protein. An overview of the assessments is provided in Table **1**.

### 2.7 Safety

Physical examination, vital signs, resting 12-lead electrocardiogram, clinical chemistry (routine blood tests, creatine kinase, renal and liver function), and urinalysis will be performed for safety evaluation.

The levels of creatine kinase and liver test analyses will be measured. Any adverse events will be reported and carefully analyzed to ensure the safety of the patients.

### 2.8 Sample Size

The primary endpoint is the change from baseline in fasting TG levels. According to results of studies published by Zhao *et al*. and Jones *et al*., TG levels decreased by 0.62 mmol/L from baseline in the xuezhikang group and 0.16 mmol/L in the pravastatin group [18, 20]. Assuming that the difference in TG reduction between xuezhikang and pravastatin was 0.46 ± 0.66 mmol/L based on existing literature [18], to achieve a similar reduction using a two-sample t test with a statistical power of 90% and a significance level of 0.05, a minimum of 45 patients must be enrolled. A total of 114 patients will be recruited after considering a dropout rate of 20%. Sample size estimation was carried out by a professional statistician using nQuery version 6.01 (Statistical Solutions, Saugus, MA).

### 2.9 Statistical Analysis

The analysis of covariance will be performed on the primary endpoint with last observation carried forward for missing values, as well as on the secondary efficacy parameters. All statistical tests between treatment groups will be tested at the 2-sided <0.05 significance level. Descriptive statistics will be performed on the safety outcomes. Two analysis sets will be used for the statistical testing: the full analysis set, including all randomized patients with baseline and at least one post-baseline data, and the per-protocol analysis set, including all randomized patients completing the study without major protocol deviations.

## 3 RESULT AND DISCUSSION

To our knowledge, this is the first randomized controlled trial to compare the TG lowering effect of xuezhikang and a synthetic statin, pravastatin, in the fasting and postprandial states of patients with T2DM and dyslipidemia without ASCVD. Xuezhikang is a lipid-lowering agent that has been proven to reduce cholesterol levels and incidence of cardiovascular events, as well as overall mortality in Chinese patients with CHD in several studies [15, 25, 26]. A systematic review and meta-analysis study that included 16 randomized clinical trials with treatments over 8 weeks showed that xuezhikang significantly reduced fasting TG levels in addition to other lipid parameters in patients with T2DM, compared with statins [17]. However, all the trials included in this systematic review were conducted in China and most of them were published in journals written in Chinese, which are not accessible to the global community, in particular, the design and methodology. More importantly, postprandial TG levels were never explored in those studies. With that in mind, our study design is planned to address the shortcomings in current studies as mentioned above.

TG control may be interrupted by fasting glucose levels [27, 28], therefore, in the present study, patients with well-controlled glucose, confirmed during the first two visits, will be enrolled. In the initial screening and after a 14-day run-in period, patients with HbA1c ≥8.0% or who have switched hypoglycemic drugs will be excluded. All patients included in this study have been diagnosed with T2DM and dyslipidemia, without ASCVD, and require primary prevention. According to Chinese guidelines for the management of dyslipidemia in adults [29], moderate-intensity statin treatment is recommended as first-line lipid-lowering therapy to reduce the risk of ASCVD. Xuezhikang (1.2 g/day) is qualified as a moderate-intensity statin (defined as LDL-C reduction of 25 to <50%) for primary ASCVD prevention in patients with T2DM [29]. Patients with very high ASCVD risk are not included in this study, as the target of LDL-C control is higher, <1.4 mmol/L and even lower [30], for which intensive statins and/or the combination of other cholesterol-lowing drugs may be recommended as the initial therapy [31, 32]. Subjects will be recommended to receive a combination therapy, such as a statin and ezetimibe, if they fail to achieve the LDL-C goal after the 6-week of monotherapy.

In this study, a high TG level is defined as fasting TG ≥1.7 mmol/L and <5.6 mmol/L. It has been reported that fasting TG levels ≥5.6 mmol/L are associated with a high risk of pancreatitis, and other TG-lowering agents such as fibrates, niacin, and omega-3 fatty acids are needed [33, 34]. Hence, patients with fasting TG ≥5.6 mmol/L will not be enrolled in this study. It is also known that fasting TG levels ≥1.7 mmol/L indicate higher risk of ASCVD although there is no TG goal recommended in the 2019 European Society of Cardiology guidelines [35]. The combination treatment of statins and fibrates reduced ASCVD events in patients with diabetes with fasting TG levels ≥2.3 mmol/L [36]. Treatment with highly purified eicosapentaenoic acid plus statins also showed cardiovascular protection, which reduced the risk of a composite of cardiovascular death, nonfatal myocardial infarction, nonfatal stroke, coronary revascularization, or unstable angina, in patients with CHD and elevated fasting TG levels (1.52-5.63 mmol/L), especially in patients with diabetes [37]. These observations support that fasting TG reduction could be beneficial for diabetic patients with hypertriglyceridemia. Therefore, the fasting TG level is designed as the primary outcome assessment in this study.

In addition, a 2019 expert panel statement pointed out that ASCVD risk is more closely related to postprandial TG levels than fasting TG levels [38]. It is recommended that the postprandial TG levels should not exceed 2.0 mmol/L (175 mg/dL) after consuming a daily meal in patients with fasting TG levels <1.7 mmol/L [39]. The elevated TG levels >2.0 mmol/L, especially in the postprandial state, contain an increase in TG-rich lipoproteins and their remnants in the circulation, the latter of which are as atherogenic as LDL [40, 41]. Thus, postprandial TG levels will also be measured after 6-weeks of treatment with xuezhikang or pravastatin.

Lp(a), similar in structure to LDL, is an atherogenic lipoprotein, and a high Lp(a) level is related to an increased residual ASCVD risk [42, 43]. Although the therapeutic target of Lp(a) has been proven to be difficult, several medicines, including proprotein convertase subtilisin/kexin 9 inhibitors (PCSK9i), have shown their effects in Lp(a) reduction [44]. PCSK9i may reduce Lp(a) concentration by enhancing its clearance and reducing its production [45, 46]. A pharmacological study showed the combination of xuezhikang and ezetimibe significantly attenuated the increase in PCSK9 level, resulting in the marked upregulation of LDL receptors, which may explain the role of xuezhikang in Lp(a) regulation [47]. The fasting Lp(a) is designed as an exploratory outcome in this study to investigate the effect of xuezhikang on the Lp(a) level. It is worth noting that the new onset of diabetes risk is a potential side effect in patients taking statins [48]. A retrospective study of red yeast rice prescriptions in Taiwan showed that xuezhikang has a lower risk in the new onset of diabetes than lovastatin, the rates of diabetes were 1.01 and 2.59 per 100 person-years, respectively [49]. To explore the impact of xuezhikang and a synthetic statin on glucose profile, fasting and postprandial glucose and insulin levels, and HbA1c level are assessed as the secondary objectives.

## 4 LIMITATIONS

There are two limitations of this study. First, the treatment duration is only 6 weeks. Second, this study will not provide information about the pharmacological mechanism of xuezhikang.

## CONCLUSION

To our knowledge, this is the first randomized controlled study to evaluate the effect of 6-week treatment with xuezhikang 1.2 g/day and pravastatin 20 mg/day on fasting and postprandial TG levels as well as other blood lipid parameters in patients with T2DM and dyslipidemia without ASCVD. The results will provide more information on how to optimize lipid control for patients with diabetes in the primary prevention of ASCVD.

## AUTHOR’S CONTRIBUTIONS

Ling Liu is the primary investigator and designer of this study. Jin Xu, Liyuan Zhu, Yingying Xie, Miao Zhang, Zixi Xiao, Rongkai Su and Tie Wen participated in the design of this study. Jin Xu and Yingying Xie drafted the manuscript. Jin Xu and Ling Liu revised the manuscript. All authors contributed to the article and approved the submitted version.

## Figures and Tables

**Table 1:** Study plan.

**Visits**	**Visit 1**	**Visit 2**	**Visit 3**	**Visit 4**
Time point (day)	-14	-1	0	42
Informed consent	×			
Inclusion/exclusion criteria	×	×		
Randomization			×	
Dietary guidance	×	×	×	×
Demographic data	×			
Previous medical history	×	×		
Physical examination	×	×	×	×
Nutritious breakfast			×	×
Fasting blood lipid routine (TC, TG, HDL-C, LDL-C)		×		
Fasting HbA1c levels		×		×
Blood lipids complete set (TC, TG, HDL-C, LDL-C, non–HDL-C, RC, Lp(a), Apo A1, ApoB) at fasting and 2 and 4 h after the meal, blood lipid routine (TC, TG, HDL-C, LDL-C) at 1 h after the meal			×	×
**High sensitivity** C-reactive protein at fasting and 2 and 4 h after the meal			×	×
Insulin levels at fasting and 1 and 2 h after the meal			×	×
Plasma glucose levels at fasting and 1 and 2 h after the meal			×	×
Blood routine, urine routine, urine pregnancy test (for fertile women)		×		×
Creatine kinase		×		×
Hepatic function test		×		×
Renal function test		×		×
Electrocardiogram		×		×
**Health education and diet instructions**			×	
×	×		×	×
Record adverse events	×	×	×	×
Distributing a snack containing carbohydrate before leaving hospital			×	×
Distributing drugs and log cards			×	
Retrieving drugs and log cards				×

**Abbreviations:** TG, triglycerides; TC, total cholesterol; HDL-C, high density lipoprotein cholesterol; LDL-C, low density lipoprotein cholesterol; non-HDL-C, non-high density lipoprotein cholesterol; RC, remnant cholesterol; Lp(a), lipoprotein (a); Apo A1, Apolipoprotein A1; ApoB, Apolipoprotein B.

## Data Availability

The data from the current study are available from the corresponding author on reasonable request.

## LIST OF ABBREVIATIONS

Apo: Apolipoprotein
ASCVD: Atherosclerotic Cardiovascular Disease
CHD: Coronary Heart Disease
HDL-C: High Density Lipoprotein Cholesterol
LDL-C: LDL-cholesterol
Lp(a): Lipoprotein(a)
PCSK9i: Proprotein Convertase Subtilisin/Kexin 9 Inhibitors
T2DM: Type 2 Diabetes
TG: Triglyceride
